# Genomic Detection of Schmallenberg Virus, Israel

**DOI:** 10.3201/eid2708.203705

**Published:** 2021-08

**Authors:** Adi Behar, Omer Izhaki, Asael Rot, Tzvika Benor, Mario Yankilevich, Monica Leszkowicz-Mazuz, Jacob Brenner

**Affiliations:** Kimron Veterinary Institute, Beit Dagan, Israel (A. Behar, O. Izhaki, A. Rot, M. Leszkowicz-Mazuz, J. Brenner);; Veterinary Field Services, Beit Dagan, Israel (T. Benor, M. Yankilevich)

**Keywords:** Schmallenberg virus, genomic detection, Culicoides, viruses, Israel, vector-borne infections

## Abstract

We discuss genomic detection of Schmallenberg virus in both *Culicoides* midges and affected ruminants during June 2018–December 2019, demonstrating its circulation in Israel. This region is a geographic bridge between 3 continents and may serve as an epidemiologic bridge for potential Schmallenberg virus spread into Asia.

Simbu serogroup viruses form one of the largest serogroups in the genus *Orthobunyavirus* of the family *Peribunyaviridae*, comprising >25 antigenically different, but serologically related, negative-sense single-stranded RNA viruses. These viruses are transmitted mainly by *Culicoides* biting midges; they persist in the environment by cycling between infected mammalian hosts and *Culicoides* vectors. Notable examples from the Simbu serogroup are Akabane virus (AKAV), Aino virus, Schmallenberg virus (SBV), Sathuperi virus (SATV), Shamonda virus (SHAV), Peaton virus (PEAV), and Shuni virus (SHUV, which is also suspected of infecting humans.). These viruses are known to cross the placenta of ruminants to the developing fetus, causing abortion, stillbirth, and neonatal malformations that are seen only at birth. The congenital malformations are termed arthrogryposis-hydranencephaly syndrome. Given that the clinical signs can be observed only months after viremia has occurred, field and laboratory practitioners are at a huge disadvantage when facing epidemics caused by these viruses (*1**–**7*).

Until recently, the most studied viruses of the Simbu serogroup were AKAV and Aino virus, both known to be present in Israel (*1*,*3*). In 2011, a new Simbu virus emerged in Europe and was named Schmallenberg virus (SBV) (*8*). Studies suggested that SBV is a reassortant virus, deriving the medium (M) RNA segment from SATV and the small (S) and large (L) RNA segments from SHAV, probably as a result of co-infection of these viruses in either *Culicoides* vectors or the ruminant hosts (*1*,*9*,*10*).

Once SBV emerged in Europe, it was clear to our team in Israel that this virus was either already present in Israel or would be introduced in the future. After AKAV and SHUV outbreaks (*3*,*11*) and virus neutralization test assays showing the additional presence of SATV, SHAV, and PEAV in Israel (*12*), a systematic monitoring system for arboviruses was established in 2015. Serum samples and vectors are collected every month from 13 selected dairy farms representing different geographic regions in Israel (Figure). Specific PEAV, SHUV, and SATV RNA fragments were also detected by nested quantitative PCR (qPCR) from different *Culicoides* species during 2015–2017 (*13*). Furthermore, in 2017, RNA fragments of a specific PEAV were detected in the cerebrospinal fluid (CSF) and testicles of a malformed calf exhibiting hydranencephaly (*14*). SBV was not found in all the studies conducted during 2011–2017, nor was it detected passively in Israel (*3*,*11**–**14*). We report the detection of SBV RNA in Israel in both vectors and affected ruminants. 

**Figure:**
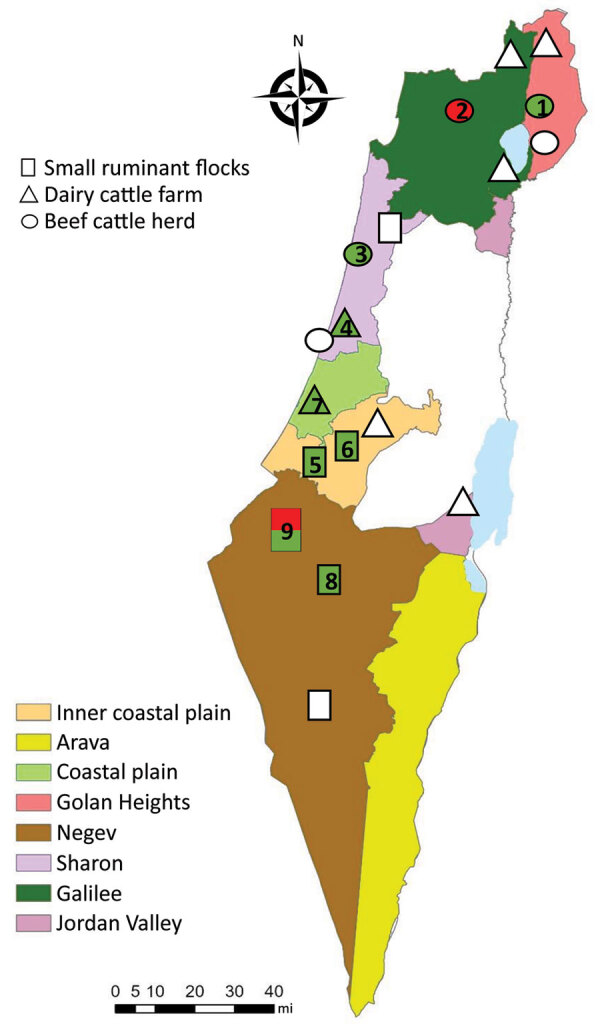
Locations and types of farms sampled in study of Schmallenberg virus (SBV), Israel. Farm number match those listed in Table 2. Green, farms from which SBV-positive *Culicoides* pools were collected; red, farms on which SBV-positive malformed progeny were detected.

## The Study

During June 2018–December 2019, we trapped 13 pools of *Culicoides imicola*, 8 pools of *C. oxystoma*, 5 pools of *C. puncticollis*, and 5 pools of *C. newsteadii* midges (each pool containing 50 midges) around livestock farms, and we tested CSF from 3 malformed 1-day-old lambs (born on July 3, 2019) and 1 malformed 11-day-old calf (born on November 1, 2019) (Figure; Appendix). We extracted RNA from *Culicoides* homogenates and CSF using Maxwell 16 Viral Total Nucleic Acid Purification Kit (Promega, https://www.promega.com) according to the manufacturer’s instructions. We used total viral nucleic acids (0.4 µg) for cDNA synthesis by UltraScript Reverse transcription (PCR Biosystems, https://www.promega.com) according to the manufacturer’s instructions. We performed reverse transcription (RT) nested qPCR targeting the L RNA segment of Simbu serogroup viruses according to Behar et al. (*13*). We further subjected samples suspected of being Simbu serogroup positive to RT-nested and seminested PCRs performed using S, M, and L segment-specific primer sets (Table 1; Appendix).

**Table 1:** Primer sets used for the amplification of Schmallenberg virus RNA-specific fragments of the S, medium M, and L segments by reverse transcription nested PCR*

Segment	External primer sequence, 5′ → 3′	Internal primer sequence, 5′ → 3′	Expected product size, bp	Reference
S	AKAI206F: CAC AAC CAA GTG TCG ATC TTA	S_nestF: TGG TTA ATA ACC ATT TTC CCC A	370	External: (*4*); internal: this study
	SimbuS637: GAG AAT CCA GAT TTA GCC CA	S_nestR: GTC ATC CAY TST TCW GCA GTC A		
M	924F: CCG AAA ACA AGG AAA TTG TG	1899F: TAT AGT CCC TGG ATT AGG TC	430	Forward primers: (*8*); reverse primers: this study
	2331R: GGT TCA AAC ATC TCT AGG C	2331R: GGT TCA AAC ATC TCT AGG C		
L	SNL_F: GCA AAC CCA GAA TTT GYW GA	panOBV-L-2959 F: TTG GAG ART ATG ARG CTA ARA TGT G	370	External: this study; internal: (6)
	SNL_R: ATT SCC TTG NAR CCA RTT YC	panOBV-L-3274R: TGA GCA CTC CAT TTN GAC ATR TC		

*L, large; M, medium; S, small.

Of the 31 species-specific pools from the 4 *Culicoides* midge species that are known or suspected to be vectors of Simbu serogroup viruses (i.e., *C. imicola*, *C. oxystoma*, *C. puncticollis*, *C. newsteadii*) (*2*,*12*,*15*), we found that 11 contained RNA of Simbu serogroup viruses in 2018 and 2019 (35% of the total pools tested) (Table 2; Figure; Appendix Table). We identified partial nucleotide sequences of the S (370/830 bp) and L (370/6,882 bp) segments. Phylogenetic analysis of the samples showed that all positive samples were virtually identical to SBV (GenBank accession nos. MT816474–82, MT816485–95) (Appendix Figure, panels A, C). These samples were collected from several different geographic regions in Israel (Table 2, lines 1 and 3–12 in the Samples column; Figure; Appendix Table). In addition, we detected SBV RNA-specific fragments of the S (370/850 bp), M (430/4,373 bp), and L segments (370/6,882 bp) in a CSF sample from a malformed lamb born in July 2019 on a farm in southern Israel (Negev desert) and a malformed calf born in November 2019 on a farm in northern Israel (Galilee) (GenBank accession nos. MT816472, MT816473, MT816483, MT816484, MT816496, MT816497) (Table 2, lines 2 and 13 in the Samples column; Appendix Figure).

**Table 2:** Samples that tested positive for Schmallenberg virus by reverse transcription nested PCR, Israel*

Geographic region	Sample source	Collection date	Infected farm type (farm no.)
Golan Heights (latitude 34.1)	*Culicoides oxystoma* midge	2018 Sep	Beef cattle (1)
Galilee (latitude 32.7–33.5)	Malformed calf	2019 Nov	Beef cattle (2)†
Sharon plain (latitude 32.2)	*C. imicola* midge‡	2018 Jun	Beef cattle (3)§
	*C. puncticollis* midge	2018 Jun	Beef cattle (3)§
	*C. newsteadii* midge	2018 Jun	Beef cattle (3)§
	*C. imicola* midge	2018 Jul	Dairy cattle (4)
Interior plain (latitude 31.89)	*C. imicola* midge‡	2018 Nov	Small ruminant farm (5)†§
	*C. imicola* midge	2018 Nov	Small ruminant farm (5)†§
	*C. imicola* midge‡	2019 Dec	Small ruminant farm (6)§
Coastal plain (latitude 31.89)	*C. oxystoma* midge	2018 Jun	Dairy cattle (7)
Negev desert (latitude 29.7–30.714)	*C. oxystoma* midge	2018 Nov	Small ruminant farm (8)§
	*C. puncticollis* midge	2019 Jul	Small ruminant farm (9)†¶
	Malformed lamb	2019 Jul	Small ruminant farm (9)†¶
South Jordan Valley (latitude 31.56)	NA	NA	NA

*NA, not applicable. †Farms on which dams and ewes gave birth to stillborn and malformed neonates. ‡Samples were confirmed positive at Friedrich Loeffler Institute, Greifswald, Germany. §Farms expecting a rate of 80%–85% prolificacy, but during calving season showed only 50%–65% prolificacy. ¶Sheep farm from which both insects and malformed lambs were sampled.

In general, the most susceptible period for induction of congenital malformations by Simbu serogroup viruses is 65–70 days of gestation in lambs and 150 days of gestation in calves (*1*,*7*). Thus, SBV detection in the respective ruminants fits with viral infection in March–April 2019, suggesting exposure to SBV in Israel in early spring 2019. Nevertheless, reports on severe decline in progeny prolificacy, stillbirths, and malformed lambs were reported by farmers to the Veterinary Field Services from autumn 2018 through December 2019 (Table 2). The detection of SBV in *Culicoides* pools collected from several of those farms (Table 2, lines 3–5, 7–9, and 11–12 in the Sample column; Figure; Appendix Table) suggests that SBV might have been clinically affecting ruminants in Israel as early as June 2018.

## Conclusions

Our results demonstrate the circulation of SBV outside Europe. Future studies are needed to determine the seroprevalence of SBV in the Middle East, because this information is essential for understanding the risk of SBV spread into countries in Asia. Because SATV is found in the Middle East (*12*,*13*), virus neutralization tests will probably not be able to properly distinguish between antibodies against SBV and those against SATV. Therefore, developing of a competitive ELISA system using SBV-specific antibodies is crucial. Finally, the presence of both SATV and SBV in Israel provides a unique opportunity for comparative studies on possible cross-protection of SBV commercial vaccines between these viruses.

Appendix Additional information on the genomic detection of Schmallenberg virus, Israel.
