# Supplementary material for: Genomic Detection of Schmallenberg Virus, Israel
Source: Emerg Infect Dis. 2021 Aug;27(8):2197–200. doi: 10.3201/eid2708.203705 (PMC8314812; doi:10.3201/eid2708.203705)
Supplement: Appendix — Additional information on the genomic detection of Schmallenberg virus, Israel. [file 20-3705-Techapp-s1.pdf]

Article DOI: https://doi.org/10.3201/eid2708.203705

Genomic Detection of Schmallenberg Virus, Israel

Appendix

Materials and Methods

Culicoides Collection

Culicoides midges were trapped on livestock farms from 7 geographic regions in Israel (Figure, main article): Negev desert (latitude 29.7–30.714086; 3 small ruminant farms), South Jordan valley (latitude 31.56; 1 dairy farm), interior plain (latitude 31.89; 1 dairy farm, 2 small ruminant farms), coastal plain (latitude 31.89; 2 dairy farms), Sharon plain (latitude 32.2; 1 dairy farm, 1 beef cattle farm, 1 small ruminant farm), Galilee (including the north Jordan valley; latitude 32.7–33.5; 2 dairy farms, 1 beef cattle farm), and Golan Heights (latitude 34.1; 1 dairy farm, 2 beef cattle farms) during June 2018–December 2019.

Culicoides midges were collected using suction light traps equipped with an 8 W blacklight and a downdraft suction motor powered by 2 rechargeable 1.5 V GP2700 AA batteries. Insects were collected into a reusable plastic jar suspended below the trap's fan. Two light traps were placed overnight (1 h before sunset and retrieved 1 h after dawn) at suitable locations on each farm, as close to the livestock as possible, suspended at a height of 1.7–2 m above the ground. Immediately after collection, the plastic jars containing live *Culicoides* midges were placed in 4°C cooling boxes and transported to the laboratory. Upon their arrival, the live insects were anesthetized with CO_2 and sorted to species under a stereoscopic microscope (Nikon SMZ25, https://www.microscope.healthcare.nikon.com) using various taxonomic keys (*1*–*4*). After sorting, live *Culicoides* midges were grouped in pools according to location and species, and all pools were stored at −80°C until testing for the presence of Simbu serogroup viruses. In total, 13 pools of *C. imicola*, 8 pools of *C. oxystoma*, 5 pools of *C. puncticollis*, and 5 pools of *C. newsteadii* (each pool contained 50 midges) were analyzed (Table 2, main article). Pooled *Culicoides* were homogenized according to Behar et al. (*5*).

Genomic Detection of SBV

A 200-µL aliquot of *Culicoides* homogenate or cerebrospinal fluid was used for total viral nucleic acid extraction with the Maxwell 16 Viral Total Nucleic Acid Purification Kit (Promega, https://www.promega.com) according to the manufacturer's instructions. The remaining 300 µL was kept at −80°C for later use. Total viral nucleic acids (0.4 µg) were used for cDNA synthesis by UltraScript Reverse transcription (PCR Biosystems, https://pcrbio.com) according to the manufacturer's instructions. cDNA synthesis and subsequent initial RT-qPCR amplifications targeting the large segments of Simbu serogroup viruses were performed using Pan Simbu primers according to Fischer et al. (*6*). However, during our work with this system, our internal quality control indicated that we got a higher frequency of false positives because of a nonspecific amplification of *Culicoides* ribosome (18s rRNA) from the *Culicoides* homogenates. Moreover, we discovered that *Culicoides* homogenates have a high inhibitory effect. Consequently, a second amplification step was added to provide higher specify to our detection systems and RT-nested qPCR was performed according to Behar et al. (*5*). All samples were run in duplicate. Reactions were performed on a CFX96 Touch Real-Time PCR Detection System (Bio-Rad, https://www.bio-rad.com) with the manufacturer-recommended PCR parameters. Samples with a cycle threshold (C_t) value <30 and melting temperature (T_m) of 73°C–81°C were suspected of being Simbu serogroup positive. For further and more accurate analysis, nested and semi nested PCRs were performed as follows.

Pan Simbu RT-PCR targeting the S RNA segment was conducted according to Hirashima et al. (*7*). The PCR products served as the template for the nested PCR with primer pairs Simbu_S_nestF and Simbu_S_nestR (Table 1) (expected product size ca. 370 bp).

Pan Simbu RT-PCR targeting the large (L) RNA segments was conducted using external primers SNL_F and SNL_R (Table 1 in main article). The PCR products served as the template for the nested PCR according to Fischer et al. (*6*) (expected product size ≈370 bp).

RT-PCR targeting the medium (M) segment specific to SBV was conducted using primers 924F according to McGowan et al. (*8*) and 2331R: 5′-GGTTCAAACATCTCTAGGC-3′. The PCR products served as the template for the semi-nested PCR using primer 1899F according to McGowan et al. (*8*) and 2331R (expected product size ≈430 bp).

Negative controls (no DNA added) were always performed in parallel. No products were obtained from these controls. Positive controls were added only to the final nested or semi-nested step to avoid contamination. All samples were amplified in a conventional PCR (SensoQuest Labcycler, https://www.sensoquest.de) with 1 μM primer and addition of 10 ng/μL bovine serum albumin (BSA) in 2X PCRBIO HS Taq mix (PCR Biosystems) for a total volume of 25 μL reaction mixture according to the manufacturer's instructions. BSA was added to improve PCR amplification because the amounts of viral nucleic acid extracted from *Culicoides* and ruminant samples were relatively low. Products were separated on a 1% (w/v) agarose gel in TAE buffer (40 mM Tris-acetate, 1 mM EDTA) and stained with SmartGlow PS (Accuris Instruments, https://www.accuris-usa.com). Negative controls (no template added) were always run in parallel. No products were obtained from these controls. All PCR products were then sequenced in both directions. Sequence chromatograms were visually inspected, verified, aligned, and annotated using Geneious Pro (Biomatters, https://www.geneious.com). Phylogenetic analysis was performed using maximum likelihood implemented in PhyML (*9*). To assess confidence in the nodes, 100 bootstrap replicates were performed. All sequences were selected for phylogenetic analysis and were deposited in GenBank under accession nos. MT816472–MT816497.

References

1. Delecolle JC. Nouvelle contribution à l'étude systématique et iconographique des espèces du genre *Culicoides* (Diptera: Ceratopogonidae) du Nord-Est de la France [dissertation]. Strasbourg, France: Université Louis Pasteur de Strasbourg; 1985 [cited 2018 Jun 30]. http://www.sudoc.abes.fr/cbs/xslt/DB=2.1//SRCH?IKT=12&TRM=107788942

2. Glukhova VM. *Culicoides* (Diptera, Ceratopogonidae) of Russia and adjacent lands. Int J Dipterol Res. 2005;16:3–75.

3. Mathieu B, Cêtre-Sossah C, Garros C, Chavernac D, Balenghien T, Carpenter S, et al. Development and validation of IIKC: an interactive identification key for *Culicoides* (Diptera: Ceratopogonidae) females from the Western Palaearctic region. Parasit Vectors. 2012;5:137. https://doi.org/10.1186/1756-3305-5-137

4. Wirth WW, Dyce AL, Spinelli GR. An atlas of wing photographs, with a summary of the numerical characters of the neotropical species of *Culicoides* (Diptera: Ceratopogonidae). Contrib Am Entomol Inst. 1988;25:1–72.

5. Behar A, Rot A, Lavon Y, Izhaki O, Gur N, Brenner J. Seasonal and spatial variation in *Culicoides* community structure and their potential role in transmitting Simbu serogroup viruses in Israel. Transbound Emerg Dis. 2020;67:1222–30. PubMed https://doi.org/10.1111/tbed.13457

6. Fischer M, Schirrmeier H, Wernike K, Wegelt A, Beer M, Hoffmann B. Development of a pan-Simbu real-time reverse transcriptase PCR for the detection of Simbu serogroup viruses and comparison with SBV diagnostic PCR systems. Virol J. 2013;10:327–35. PubMed https://doi.org/10.1186/1743-422X-10-327

7. Hirashima Y, Kitahara S, Kato T, Shirafuji H, Tanaka S, Yanase T. Congenital malformations of calves infected with Shamonda virus, Southern Japan. Emerg Infect Dis. 2017;23:993–6. PubMed https://doi.org/10.3201/eid2306.161946

8. McGowan SL, La Rocca SA, Grierson SS, Dastjerdi A, Choudhury B, Steinbach F. Incursion of Schmallenberg virus into Great Britain in 2011 and emergence of variant sequences in 2016. Vet J. 2018;234:77–84. PubMed https://doi.org/10.1016/j.tvjl.2018.02.001

9. Dereeper A, Guignon V, Blanc G, Audic S, Buffet S, Chevenet F, et al. Phylogeny.fr: robust phylogenetic analysis for the non-specialist. Nucleic Acids Res. 2008;36(Web Server):W465–9. PubMed https://doi.org/10.1093/nar/gkn180

Appendix Table. Viral genomic detection in field-collected *Culicoides* species in 2018 and 2019, Israel*

Geographical region	Date	Farm type	*Culicoides imicola*		*Culicoides oxystoma*		*Culicoides puncticollis*		*Culicoides newsteadii*	
			(n/+)	Virus genome detected	(n/+)	Virus genome detected	(n/+)	Virus genome detected	(n/+)	Virus genome detected
Golan Heights	2018 Sep	Beef cattle (1)†	0	NA	1/1	SBV	0	NA	0	NA
	2019 Sep	Beef cattle	0	NA	1/0	NA	0	NA	0	NA
Galilee	2018 Sep	Dairy	0	NA	1/0	NA	0	NA	0	NA
	2019 Oct	Dairy	1/0	NA	0	NA	0	NA	0	NA
Sharon plain	2018 Mar	Small ruminant	0	NA	0	NA	0	NA	1/0	NA
	2018 Jun	Beef cattle	1/0	NA	1/0	NA	0	NA	0	NA
	2018 Oct	Beef cattle (3)	1/1	SBV	1/0	NA	1/1	SBV	1/1	SBV
	2018 Jul	Dairy (4)	1/1	SBV	1/0	NA	1/0	NA	0	NA
	2019 Nov	Beef cattle (3)	2/2	Aino, AKAV	0	NA	1/1	Aino	0	NA
Interior plain	2018 Nov	Small ruminant (5)	2/2	SBV	0	NA	0	NA	0	NA
	2018 Oct	Dairy	0	NA	0	NA	0	NA	1/0	NA
	2019 Sep		0	NA	0	NA	0	NA	1/0	NA
	2019 Dec	Small ruminant (6)	0	NA	0	NA	0	NA	0	NA
Coastal plain	2018 Jun	Dairy (7)	1/0	NA	1/1	SBV	0	NA	0	NA
South Jordan valley	2018 Mar	Dairy	1/0	NA	0	NA	0	NA	0	NA
	2019 Sep		0	NA	0	NA	1/0	NA	0	NA
Negev	2018 Nov	Small ruminant	1/1	SBV	0	NA	0	NA	1/0	NA
	2018 Nov	Small ruminant (8)	1/1	SBV	1/0	NA	0	NA	0	NA
	2019 Jul	Small ruminant (9)	1/0	NA	0	NA	1/1	SBV	0	NA

*Aino, Aino virus; AKAV, Akabane virus; NA, not applicable; SBV, Schmallenberg virus.
†Numbers refer to affected farms, related to those in Table 2 and Figure, main article. (In farm 2, *Culicoides* were not collected. Only ruminant samples were collected.)

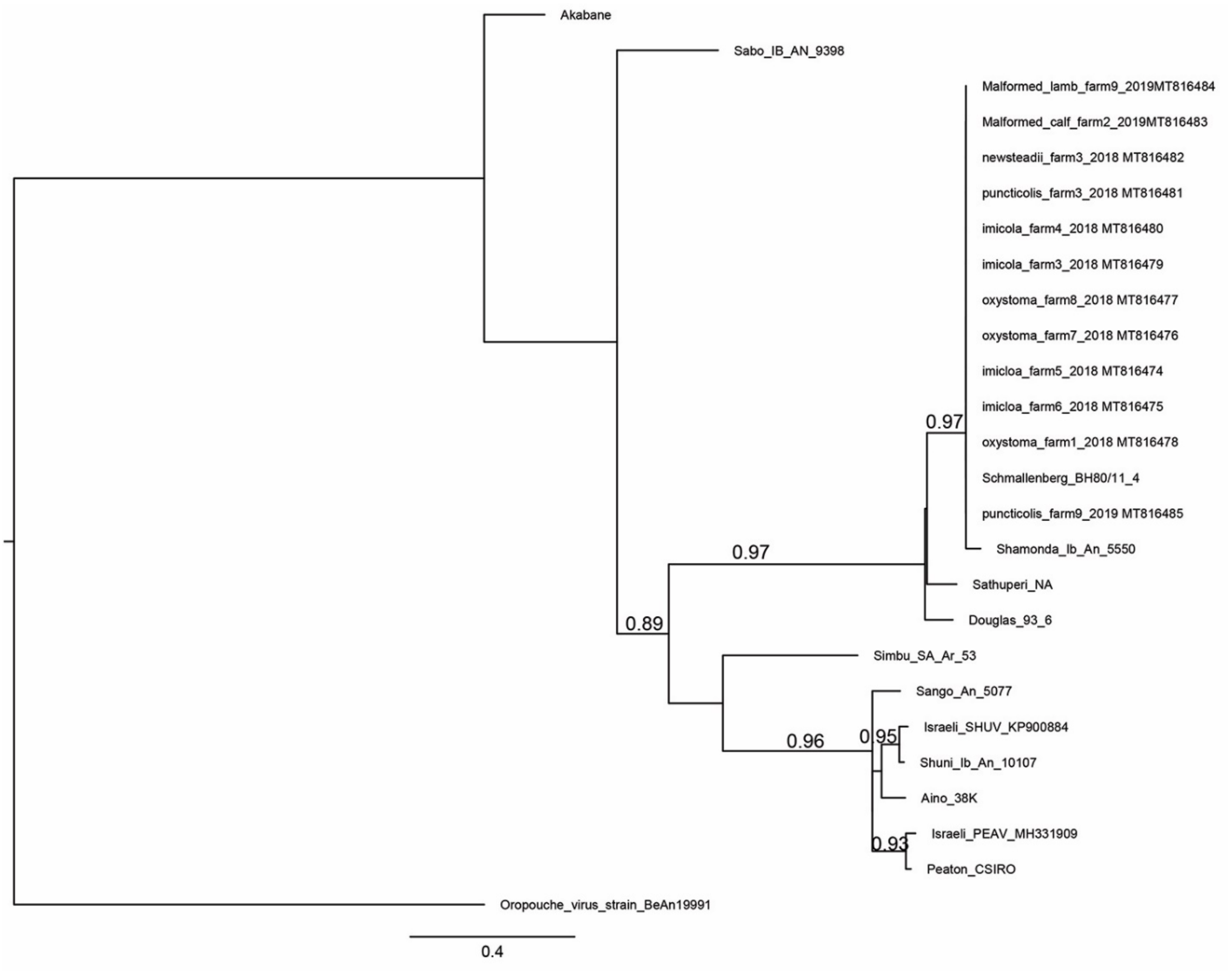
Akabane
Sabo_IB_AN_9398
Malformed_lamb_farm9_2019MT816484
Malformed_calf_farm2_2019MT816483
newsteadii_farm3_2018 MT816482
puncticolis_farm3_2018 MT816481
imicola_farm4_2018 MT816480
imicola_farm3_2018 MT816479
oxystoma_farm8_2018 MT816477
oxystoma_farm7_2018 MT816476
imicloa_farm5_2018 MT816474
imicloa_farm6_2018 MT816475
0.97
oxystoma_farm1_2018 MT816478
Schmallenberg_BH80/11_4
puncticolis_farm9_2019 MT816485
0.97
Shamonda_Ib_An_5550
Sathuperi_NA
0.89
Douglas_93_6
Simbu_SA_Ar_53
Sango_An_5077
Israeli_SHUV_KP900884
0.96
0.95
Shuni_Ib_An_10107
Aino_38K
Israeli_PEAV_MH331909
0.93
Peaton_CSIRO
Oropouche_virus_strain_BeAn19991
0.4

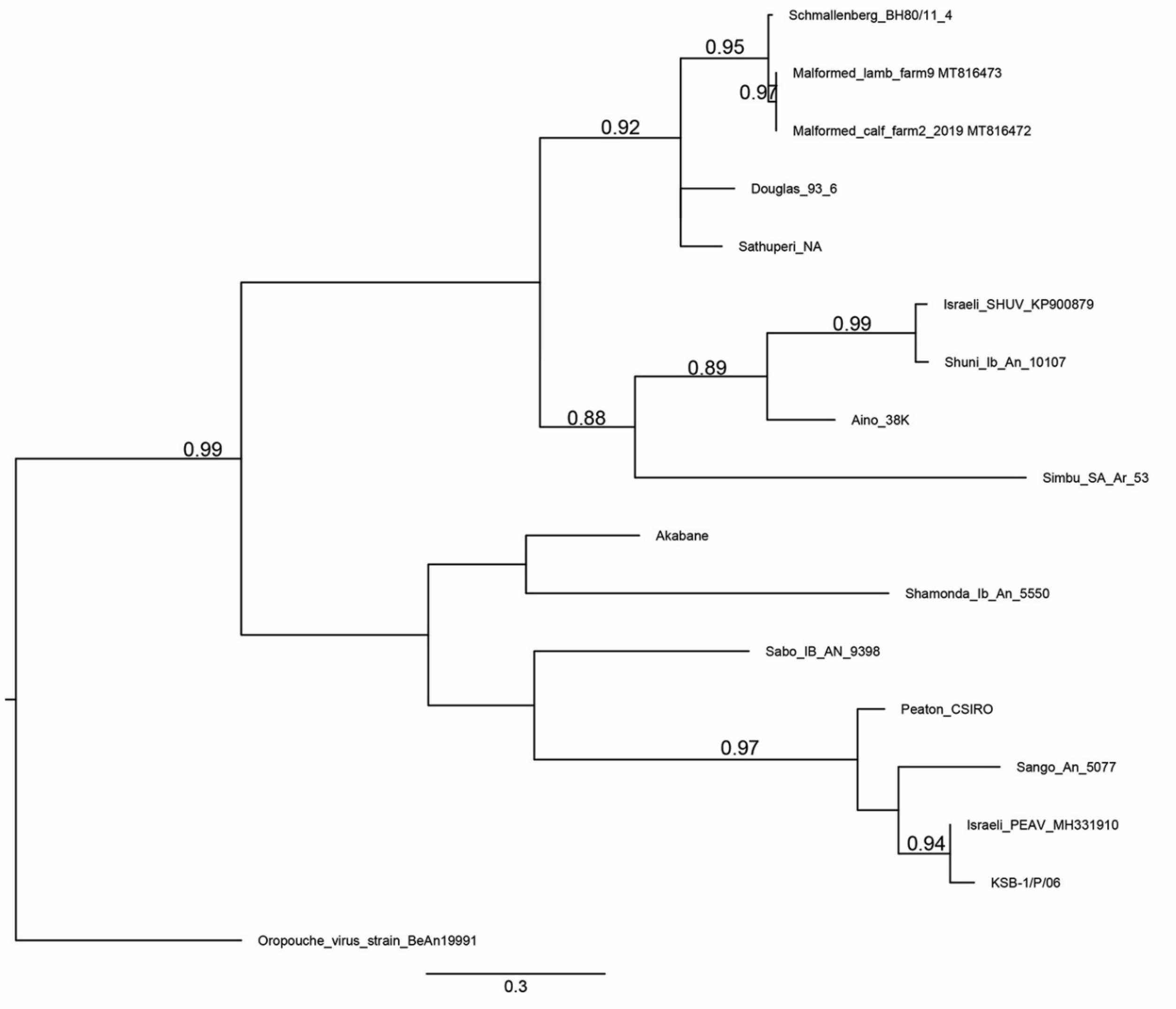
Schmallenberg_BH80/11_4
0.95
Malformed_lamb_farm9 MT816473
0.97
Malformed_calf_farm2_2019 MT816472
0.92
Douglas_93_6
Sathuperi_NA
Israeli_SHUV_KP900879
0.99
Shuni_Ib_An_10107
0.89
Aino_38K
0.88
0.99
Simbu_SA_Ar_53
Akabane
Shamonda_Ib_An_5550
Sabo_IB_AN_9398
Peaton_CSIRO
0.97
Sango_An_5077
Israeli_PEAV_MH331910
0.94
KSB-1/P/06
Oropouche_virus_strain_BeAn19991
0.3

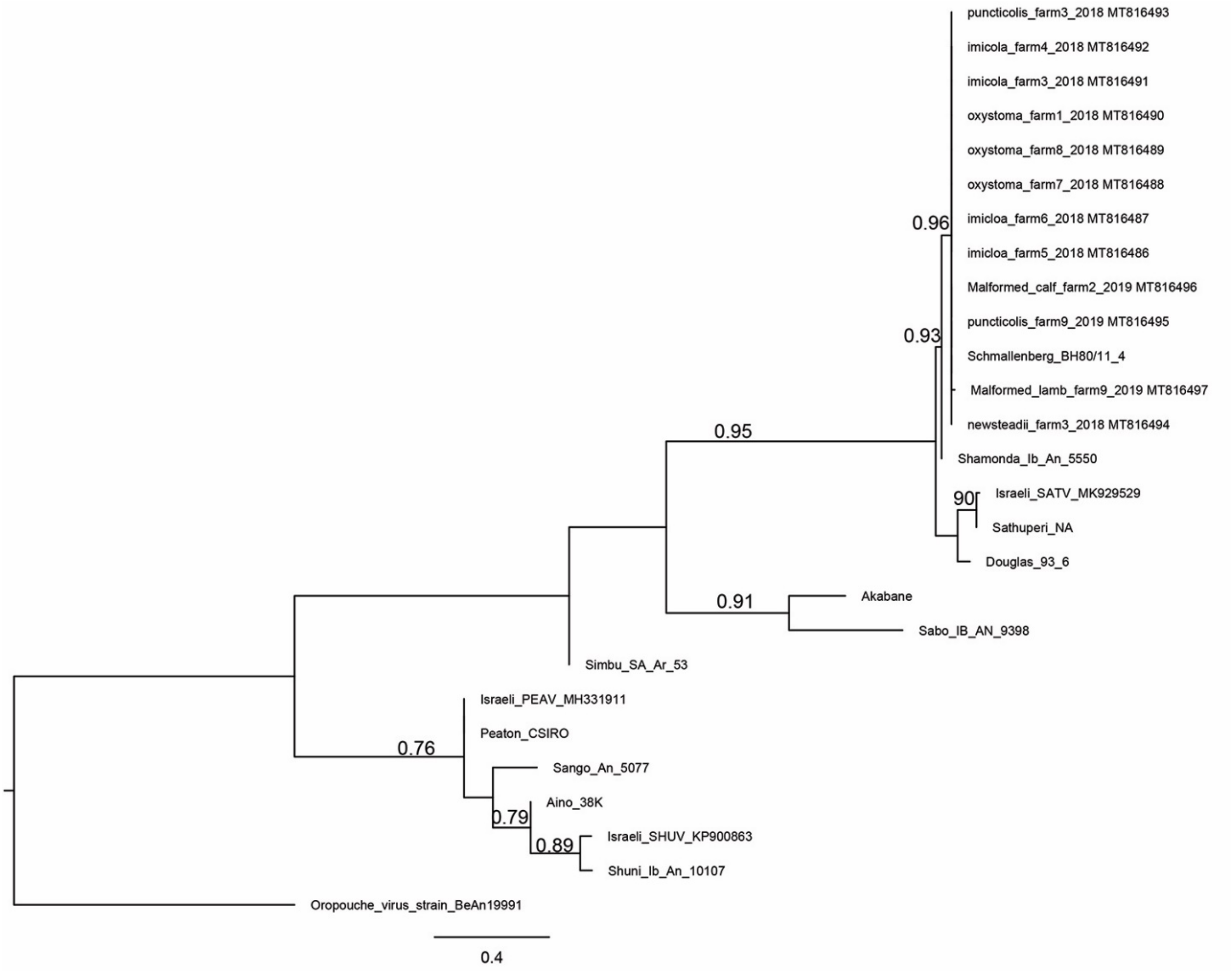

Appendix Figure. Rooted maximum-likelihood phylogenetic tree of Simbu serogroup for the (A) S, (B) M, and (C) L segments, based on a general time-reversible and gamma-distributed rate heterogeneity (GTR_G) model of nucleotide substitution. Sequences of SBV detected in positive pools of *C. imicola*, *C. oxystoma, C. newsteadii*, and *C. puncticollis* collected from different geographic regions in Israel were compared with previously published sequences of specific Peaton virus (PEAV), Shuni virus (SHUV), and Sathuperi virus (SATV) RNA fragments obtained in Israel during 2014–2017, and with the respective validated whole-genome sequences of Simbu serogroup viruses. Whenever possible, we used Simbu serogroup viruses for which full-segment sequences were available. Homologous sequences from Oropouche virus were used as the outgroup. Scale bar indicates estimated nucleotide substitutions. Only bootstrap values greater than 70% are shown.
